# Community Eye Health MSc dissertations

**Published:** 2008-12

The seven Exchange articles that follow are based on the dissertations of students at the International Centre for Eye Health, London School of Hygiene and Tropical Medicine, who graduated in 2008.

**Figure:**
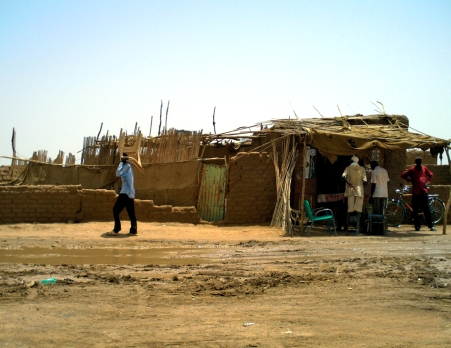
The Jabel Awliya camp for internally displaced persons (IDPs). SUDAN

