# Supplementary material for: Community Eye Health MSc dissertations
Source: Community Eye Health. 2008 Dec;21(68):64–6. (PMC2643037)
Supplement: Related texts

# Other: Situation analysis of ophthalmic services in displaced persons camps surrounding Khartoum, Sudan

**Published:** 2008-12

**Authors:** Lina Osman

**Affiliations:** Ophthalmology Trainee, Clayton Hospital, Mid Yorks NHS Trust, WF1 3JS, UK. Email: lina_a_mustafa@yahoo.co.uk

Following decades of civil war, approximately two million internally displaced persons (IDPs) are living in and around Khartoum, the capital city of Sudan; 400,000 have settled in the four official camps, and the rest live in 30 ‘squatter areas’ scattered around the city. These communities are poor, vulnerable, and at greater risk of avoidable or preventable blinding eye disease.

Our study aimed to evaluate the distribution, availability, and accessibility of eye care services in the camps for displaced persons. This descriptive situation analysis of human resources and infrastructure is a necessary first step towards providing adequate and sustainable services.

All four official IDP camps surrounding Khartoum were included in our situational analysis. Quantitative data on human resources and infrastructure was collected, using a checklist, from the ministry of health and from the available services in the visited camps. Qualitative data aimed to explore the behaviour of IDPs in seeking eye care; they included focus-group discussions with mothers of school-aged children, semistructured interviews with functionally blind IDPs, and semi-structured interviews with health care staff working in the camps.

Virtually no eye care services were found in the camps. The only permanent service found in all the visited camps was a single eye clinic in a camp housing around 150,000 people, which was integrated within a primary health care unit. When assessing the IDPs' barriers to accessing medical eye care, we found that the main ones were: poverty, the absence of services, the lack of an accompanying individual, the fear of surgery, and customs and beliefs. Given the absence of services and appropriate health care cadres, as well as the inability of IDPs to afford even subsidised basic eye drops, existing health care staff felt inadequate because they could provide very little help.

In conclusion, future service planning in the area should be directed towards affordable eye care services for the IDPs. Health service planners also need to look into the reasons behind the absence of service provision: lack of commitment, funding, or personnel.

**Figure:** An interview in an IDP camp. SUDAN

# Other: Suppurative keratitis at Groote Schuur Hospital, South Africa: epidemiology, clinical features, and microbiology

**Published:** 2008-12

**Authors:** Nicky Cockburn

**Affiliations:** Ophthalmologist, Groote Schuur Hospital, Observatory, Cape Town, South Africa. Email: nicole.cockburn@uct.ac.za

The visual prognosis of suppurative keratitis (SK) in the developing world is extremely poor, making this disease a significant cause of monocular blindness. Current standard practice in managing SK is empiric therapy with topical antibiotics, followed by a modification of this therapy based on clinical response and on microbiological results of corneal scrapings. Empiric therapy needs to be based on a knowledge of likely aetiological organisms, as these vary significantly both geographically and with time.

The aim of this study was to describe the epidemiology, clinical presentation, and microbiology of SK at Groote Schuur Hospital, in Cape Town, as recent data of this nature is not available in South Africa or Southern Africa. We conducted a retrospective review of clinical records of all patients admitted for inpatient therapy (186 patients, 210 admissions) over a three-year period (2005–2007).

The most commonly identified risk factors for SK were: neurotrophic cornea (28% of risk factors), ocular surface disease (23%), and trauma (21%). Trauma (p=0.004) and retroviral disease (p=0.001) were more commonly identified in the under-60 age group, and ocular surface disease (p=0.0001) in people over 60. Retroviral disease was more common in women (p=0.009) and trauma more common in men (p=0.0001).

Visual acuity in the affected eye was <6/60 for 78% of patients on admission and for 44% on final follow-up. Binocular acuity was <6/60 for 13% of patients on admission and for 5% on follow-up. Thirty per cent of patients underwent acute surgery on one or more admissions.

Cultures were positive for 75% of specimens. Bacteria were isolated in 89% of these, fungi in 6%, and both bacteria and fungi in 5%. Gram-positive organisms constituted 69% of all bacteria isolated. The sensitivity of Gram staining (proportion of actual positives which are correctly identified as such) for cases of bacterial keratitis in this setting was low (27%) and for fungal microscopy even lower (19%).

In conclusion, SK is a blinding condition in Cape Town, with patients presenting with advanced disease and frequently requiring acute surgical intervention. The spectrum of organisms isolated was very similar to that in other temperate regions. In this study, microscopy (bacterial and fungal) had a very low sensitivity, which is concerning.

# Other: Vitamin A defciency in Thatta District, Sindh Province, Pakistan

**Published:** 2008-12

**Authors:** Muhammad Fawad

**Affiliations:** Ophthalmologist, Aga Khan University Hospital, Karachi, PO Box 3500, Pakistan. Email: umarfawad@yahoo.com

Corneal blindness due to vitamin A deficiency (VAD) is a leading cause of preventable blindness in children in developing countries. This study aimed to evaluate whether VAD is a public health problem in children aged 6–72 months in the rural and underprivileged Thatta District, in Sindh Province, Pakistan. Another aim was to identify risk factors for VAD, and to determine the coverage of distribution of high-dose vitamin A.

Purposive sampling was used to identify high-risk areas. The caregivers of children were interviewed to collect information about demographics and risk factors of VAD. Both eyes of children were examined using torch and magnifying loupe. Height, weight, and mid upper-arm circumference were measured. A blood sample was obtained from every tenth child and clinical case in order to measure serum retinol levels, using high performance liquid chromatography.

**Figure:** Ocular examination in preschool children. PAKISTAN

**Figure:** Measuring height to assess nutritional status. PAKISTAN

Out of the 619 children examined, 18 (2.9%, 95% CI 1.58–4.22) were xerophthalmic. Mean serum retinol was 27.56 *μ*g/dl (n=49, SD +9.57) and the median was 25.64 *μ*g/dl (range=10.16–53.19 *μ*g/dl). Eight children (16.32%) had serum retinol <0.7 *μ*mol/L (20 *μ*g/dl) showing moderate subclinical VAD.

Five hundred and ninety-eight (96.6%) children had received vitamin A supplementation and 504 (81.4%) were immunised against measles.

Two hundred and twenty-seven (36.7%) mothers had night blindness (a symptom of VAD) during a recent or last pregnancy. In many locations, women thought this was a normal phenomenon of pregnancy.

Univariate analysis for individual risk factors was performed, but only age group was statistically significant (x^2^=11.97, p=0.0001), as xerophthalmia rates increased with age.

Despite high vitamin A coverage, VAD is a public health problem in preschool children and pregnant women in selected underprivileged rural areas of Pakistan. Malnutrition is widespread and levels of illiteracy among mothers were extremely high.

More studies are required with adequate sample size to identify associated risk factors. Health education and promotion activities should be run in rural areas of Pakistan to increase awareness regarding night blindness.

# Other: Childhood blindness: piloting the key informant method in Lorestan Province, Iran

**Published:** 2008-12

**Authors:** Hessom Razavi

**Affiliations:** Senior House Officer in Ophthalmology, London House, Mecklenburgh Square, London WC1N 2AB, UK. Email: hessom.razavi@gmail.com

There are very few data on childhood blindness in Iran, a lower-middle-income country with a population of around 70.5 million people. There is currently no national programme for the prevention of childhood blindness.

Our research team undertook to pilot the key informant method in three out of the nine counties in the province of Lorestan. The aims of our study were, firstly, to establish the feasibility of a key informant survey in Iran and, secondly, to provide estimates of the prevalence and causes of childhood blindness in the area.

Around 120 community health workers were trained by the author to act as key informants, to identify and refer blind children from their own communities. Two ophthalmologists then examined the children to verify that they were blind and to diagnose the cause of blindness.

Our study confirmed the feasibility of a key informant survey in Iran: the method was time- and cost-efficient, it was well received by local health authorities, and it produced credible estimates of blindness.

**Figure:** Training session on the key informant method. IRAN

Our findings were consistent with previous research conducted in Iran and in the Middle-Eastern Crescent. Out of around 110 children referred for examination, a total of 27 blind children were recruited, yielding an estimated prevalence of 0.06%, so that around 10,800 children were estimated to be blind in Iran.

The leading cause of blindness was retinal disorders (40.7%), followed by disorders of the whole globe (33.3%). The majority of cases had a hereditary aetiology (70.4%), which showed a significant association with family history of blindness (p=0.002). The vast majority of blind children had a history of parental consanguinity (92.6%). Importantly, almost 50% of the cases were potentially avoidable, and 45.8% of blind school-aged children were not attending school.

Given the encouraging outcomes of this pilot study, the next rational step is to conduct a full-scale, district-level key informant survey in Lorestan. This will contribute to the planning of a national programme for the prevention of childhood blindness in Iran. Our recommendations also included the support of ongoing work in genetic counselling and of family planning and maternal and child health services.

# Other: Incidence of blindness and low vision in Bauchi State, Nigeria

**Published:** 2008-12

**Authors:** Mohammed Abdull

**Affiliations:** Ophthalmologist and Head of Department, Department of Ophthalmology, State Specialist Hospital, PMB 005, Bauchi, Bauchi State, Nigeria. Email: abdullmm@yahoo.com

Incidence figures, i.e. the number of new cases arising during a specific period of time, are important for the long-term planning of eye care programmes.

We conducted a follow-up after three years of all the subjects aged 40 years and above seen during the 2005 national blindness and low vision baseline survey in Bauchi State, Nigeria. Our aim was to assess the three-year incidence of blindness, low vision (visual acuity (VA)<6/18–3/60), and cataract, as well as the three-year progression of cataract in this State.

Original clusters and subjects were traced and identified using enumeration records. Presenting VA was measured using the reduced LogMar chart and, for those with VA<6/12, VA was then measured using a pinhole. For all subjects, lens opacity was graded using the Mehra Minassian classification; for those with VA<6/12, pupillary dilation was performed and lens opacity assessed using the simplified WHO grading system as in the baseline survey.

We found that the three-year cumulative incidence of blindness in persons was 5.50% (95% CI 2.34–8.66), while that of low vision and blindness together was 19% (95% CI 13.56–24.44). Most of low vision was due to uncorrected refractive error. Of those who were normal at baseline, two subjects (1.19%) became blind, and 27 (16.16%) developed new low vision. Among those with low vision at baseline, nine (27.27%) new cases of blindness developed. The three-year cumulative incidence of bilateral cataract blindness was 5.33% (95% CI 1.94–11.96). The three-year progression of lens opacities was similar for nuclear and cortical opacities, at 2.39% and 2.99% respectively. The three-year mortality in the cohort was 10.3% and there was no gender difference or relationship with visual status at baseline.

Response rate was best in clusters where there had been some service provision or contact since the baseline survey.

# Other: Assessing the use of traditional eye medicines in Bukavu ophthalmic district, Democratic Republic of Congo

**Published:** 2008-12

**Authors:** Théodore Kadima Mutombo

**Affiliations:** Medical Advisor, CBM — Central Africa Regional Office; Medical Director, Celpa Eye Clinic, BP 1247 Bukavu, Democratic Republic of Congo. Email: theodorekadima@yahoo.fr

This study aimed to describe the extent of the use of traditional eye medicines (TEM) in the Bukavu ophthalmic district, in the Democratic Republic of Congo, with a view to collaborating with traditional healers in eye care promotion at community level.

A descriptive cross-sectional study of 470 consecutive patients who attended eye centres was carried out. This was combined with a qualitative study. Non-probabilistic convenience sampling was used to collect quantitative data, through a questionnaire and a clinical examination of patients. Qualitative information was gathered through in-depth interviews with six traditional healers.

**Figure:** Interviewing a traditional healer. DEMOCRATIC REPUBLIC OF CONGO

Eighty-four (17.90%) patients reported using TEM for the current disease episode before attending eye care services. There was no significant association for age, gender, or place of residence. However, the level of education (primary school and below) was significant for association with usage of TEM.

The provider of TEM was in 72.6% of cases a relative, friend, or acquaintance (‘non-professional healer’) and in 27.4% of cases a professional traditional healer (affiliated with the Congolese Association of Healers).

Among the 84 subjects who reported using TEM, 46.4% stated preference as the reason for this choice, 33.3% reported proximity as the reason, and 15.50% reported cost. No patient declared a lack of awareness of the existence of eye care services in the district.

Amongst users, TEM was used for the following conditions: 34.5% used it for acute conditions (conjunctivitis, corneal ulcers), 22.7% for chronic loss of vision (cataract and glaucoma), and 42.8% for trauma and posterior segment disease. People who chose to use TEM presented late at the hospital. 19% of those who had used TEM were blind (visual acuity <3/60) compared to 8.8% of non-users.

In conclusion, the health education of the population and the integration of traditional healers into primary eye care programmes are critical for reducing the harmful effects of traditional eye medicine. The healers interviewed expressed the willingness to collaborate with the existing eye care programme. However, much of the use of traditional eye medicine is not due to healers, but to the home practices in the region. If we are to solve this problem, the cost of services remains a key barrier to address.

# Other: Health-seeking behaviour and cost of treatment for the families of children with cataract attending Instituto Brasileiro de Oftalmologia, Brazil

**Published:** 2008-12

**Authors:** Claudia Leite

**Affiliations:** Ophthalmologist, Instituto Brasileiro de Oftalmologia, Rio de Janeiro, Brazil. Email: claudoctor@hotmail.com

Our study aimed to determine the health-seeking behaviour of families of children presenting with congenital and developmental cataract, as well as the cost of treatment to families - about which little is known.

The study was undertaken in the *Instituto Brasileiro de Oftalmologia* (IBOL) a private hospital in Rio de Janeiro, Brazil. *Instituto Catarata Infantil* (ICI), a non-governmental development organisation (NGDO), provides financial support for the treatment of children with cataract in IBOL.

**Figure:** A young cataract patient after surgery. BRAZIL

We reviewed medical records of consecutive eligible children and interviewed their caregivers using a pre-tested questionnaire, to collect information about their health-seeking behaviour and socioeconomic status, and to calculate out-of-pocket costs for treatment. We also ascertained what the NGDO covered, to find out the extent to which it was meeting out-of-pocket expenses.

Data from 70 children were gathered, 42 (60.0%) of whom had bilateral disease. Fifty-eight cases (82.9%) were considered congenital and 12 (17.1%) developmental. Late detection of cataract occurred in 20 cases (28.6%). Children with developmental cataract were 4.8 times (CI 1.3–17.8) more likely to be detected late compared to those with congenital cataracts (p= 0.018). Late presentation for treatment (treatment starting in the fourth month or later after recognition) was observed for 33 (47.1%) children. Having insurance (adjusted OR 0.17, 95% CI 0.04–0.82) and being the only child (adjusted OR 0.16, 95% CI 0.04–0.69) decreased the likelihood of late presentation.

The total annual cost per eye varied from US $7.9 to 5,540.3, and the median was 327.9 (LQ 72.2, UQ 1438.1). The median expenditure incurred by parents supported by the NGDO was approximately one-third of that by parents with total and partial insurance coverage (x^2^=43.941, p=0.0001). In total, ICI spent US $49,400 assisting 35 children, a median cost of US $415.5 (range 0–799.2, LQ 175.2, UQ 547.1) per eye per year.

In conclusion, delayed detection and presentation for treatment of non-traumatic paediatric cataract are still significant problems in the state of Rio de Janeiro. Strategies should be implemented to ensure early recognition, prompt referral, and appropriate treatment, especially in the public health sector. The cost of managing cataract in childhood is high and NGDOs play an important role in bridging financial barriers in this setting.
